# The Role of Edible Insects in Rural Livelihoods, and Identified Challenges in Vhembe District, Limpopo, South Africa

**DOI:** 10.3390/resources10120123

**Published:** 2021-12-07

**Authors:** Zabentungwa T. Hlongwane, Rob Slotow, Thinandavha C. Munyai

**Affiliations:** School of Life Sciences, University of KwaZulu-Natal, Private Bag X01, Scottsville 3209, South Africa

**Keywords:** rural livelihood, food security, income, employment opportunity

## Abstract

Edible insects are an important natural commodity in rural areas that is used for household consumption and to generate income through trade. As a result, edible-insect trading is a profitable business that provides employment and improves the livelihoods of impoverished rural people. This study aimed at determining the socioeconomic benefits of and reasons for trading insects, and to assess if edible insects are included in economic development strategies in the Vhembe district of Limpopo province, South Africa. We conducted 72 questionnaire interviews targeting traders in 5 towns across the district. Five insect groups belonging to four insect orders are traded in informal markets of the district. Mopane worms (*Gonimbrasia belina*) were the most traded (42%) edible insects. Unemployment (45%) and the demand for edible insects (34%) were the major reasons for trading insects. Insect trading has numerous benefits; however, the provision of income (60%) and financial support (35%) were stated as the primary benefits. Despite several benefits associated with trading in insects, there are many challenges such as insect spoilage and a decline in the availability of edible insects in the wild. Edible insects play an important role in food security and the rural economy by generating employment opportunities for unemployed traders. Trading in insects is a traditional practice based on indigenous knowledge, which has persisted as an economic practice that improves rural livelihoods by reducing poverty and increases the human dignity of rural citizens. Only four governmental organisations in Limpopo included edible insects in economic development strategies. Trading insects is primarily an informal activity. The government could stimulate the activity and broaden and deepen the community benefits by providing infrastructure, access to harvest areas, financial support, and business training as part of a rural empowerment strategy to end hunger and poverty while creating employment opportunities in rural areas.

## 1 Introduction

The majority of rural populations in southern and eastern Africa are food-insecure and susceptible to malnutrition [1]. In addition, rural communities in Africa often experience high poverty levels that exacerbate malnutrition and food-insecurity problems [1–3]. Rural areas in South Africa are underdeveloped, and job opportunities are scarce [4]. As a result, most rural populations are poor and depend on cash handouts from the government in the form of social grants and the subsistence use of natural resources for survival [4]. The use of wild natural resources such as edible insects is a rural safety-net strategy against poverty and hunger [5].

Rural communities in developing countries are greatly dependent on indigenous natural resources, such as wild fruits and edible insects, to sustain their livelihoods [1,2,5–8]. Natural resources from the wild play an essential role in rural livelihoods in various communities in South Africa [9]. These natural resources are primarily used for household consumption and to generate income through trading [5,7,9]. As a result, wild fruits and edible insects play an important role in food security in rural areas [2,6]. They are also used as an alternative source of nutrition [2,10].

The consumption of insects is a common practice in South Africa, particularly in the Limpopo, Mpumalanga, Gauteng, and North West provinces [10,11]. Edible insects are consumed because they play a significant traditional role in nutritious diets in various parts of South Africa [11,12] as an important source of protein, vitamins, fats, amino acids, and minerals [10,13,14]. As a result, edible insects are regularly consumed or used to supplement diets during times of hardship and when staple food is scarce [15,16].

Edible-insect trading is a good business that generates income and improves livelihoods in rural communities. The generated income from edible insects varies from species to species. For instance, *Gonimbrasia belina* (mopane worm) is an economically important caterpillar in southern Africa, which plays a vital role in rural income generation [17–19]. Approximately 16,000 metric tons of mopane worms are traded per year, resulting in an income generation of about USD 39 million to 59 million a year in southern Africa [20]. Mopane worm traders in the town of Thohoyandou in the Limpopo province of South Africa can each generate an income of approximately USD 1393 (about ZAR 21,060) per year [21]. Other economically important species include termites, which are widely consumed across Africa, creating earning opportunities for rural traders and harvesters [12,22,23].

The edible-insect trade could become an excellent commercial business with significant economic returns that can improve the rural economy and create job opportunities [24]. It could hugely improve rural livelihoods, food security, and human nutrition in South Africa, and can be used as a strategy to alleviate poverty in poor communities [5,24]. Despite the significant role of edible insects in improving rural livelihoods, there is still no clear policy and legislation that governs edible-insect trading and consumption in South Africa. There is also limited information on policy documents and regulations that support and promote edible insects as food. Therefore, this study determines the socioeconomic benefits of trading insects in the Vhembe district, South Africa, and the associated reasons for trading insects by: (1) assessing if edible insects are included in economic development strategies in the Vhembe district of Limpopo Province of South Africa; (2) identifying economically important edible insect groups; and (3) determining the benefits and monetary value associated with trading edible insects. On the basis of our results, we provide policy recommendations to improve rural livelihoods and wellbeing.

## 2 Materials and Methods

### 2.1 Study Area

The study was conducted in December 2019 across six towns in the Vhembe district of Limpopo province in South Africa: Elim (23.1561° S 30.0554° E), Makhado (23.0462° S, 29.9047° E), Sibasa (22.9325° S, 30.4674° E), Thohoyandou (22.8785° S, 30.4818° E), Musina (22.3813° S, 30.0319° E), and Biaba (22.540° S, 30.0319° E).

A questionnaire comprising open-ended and closed questions was used to source information from traders in each town, selected on the basis of our observations of people buying and selling insects in the markets, bus terminals, taxi ranks, and along roadsides. The questionnaires were administered through structured face-to-face interviews with the traders. All interviews were individually conducted in the Tshivenda language (one of the 11 official languages in the South Africa). The surveyed number in each town was based on the scale of the trade that we observed, and we interviewed as many individuals as possible. Traders were asked to list the number of species that they were selling, and where they harvested or bought their insects for trading, to describe how they processed the insects, the reason for selling insects, socioeconomic benefits, and associated challenges with selling insects (Supplementary File S1).

The assessment of trading conditions considering food safety, hygiene, trading structure, shelter, storage, safety, sanitation and water availability was conducted across trading places in Vhembe district.

This study has been ethically reviewed and approved by the University Human and Social Sciences Research Ethics Committee (approval number HSS/0125/019D). Permission to conduct research in various towns was obtained from the Vhembe district municipality. All participants provided informed consent to participate in the study, and data were anonymised, confidentially treated, and securely stored.

### 2.2 Data Analysis

Data from the questionnaires were coded and entered into a Microsoft Excel spreadsheet. A chi-squared test of independence was used to determine if there were any significant differences in the number of insect species harvested and sold across small towns. The count data of respondents are presented in percentages. Chi-squared test analysis was performed using IBM SPSS Statistics version 26 (SPSS Inc., Chicago, IL, USA).

## 3 Results

A total of 72 informal street vendors selling one or more species of edible insects were surveyed in the Vhembe district, Limpopo. These traders were from the towns of Thohoyandou (27), Tshakhuma (14), Sibasa (10), Musina (7), Louis Trichardt (6), Elim (6), and Biaba (2). The majority of traders were women (94%) between the ages of 18 to 74 years, while only 6% of traders were males between 35 and 65 years old (Table A1). Most traders (30%) were between 35 to 44 years old, followed by 45 to 54 years (22%), and the fewest traders (8%) were young people between the ages of 18 to 24 years (Supplementary File S1).

The educational background of traders ranged from no formal education to tertiary education, with the majority of traders (65%) having obtained secondary education, followed by primary education (22%), and the fewest (3%) traders having had tertiary education. The majority of traders (98%) were unemployed and made a living by selling edible insects, while only 2% of the traders were employed and selling insects part-time. In addition, 97% of the traders were breadwinners in their households (Table A1).

**Figure 1:**
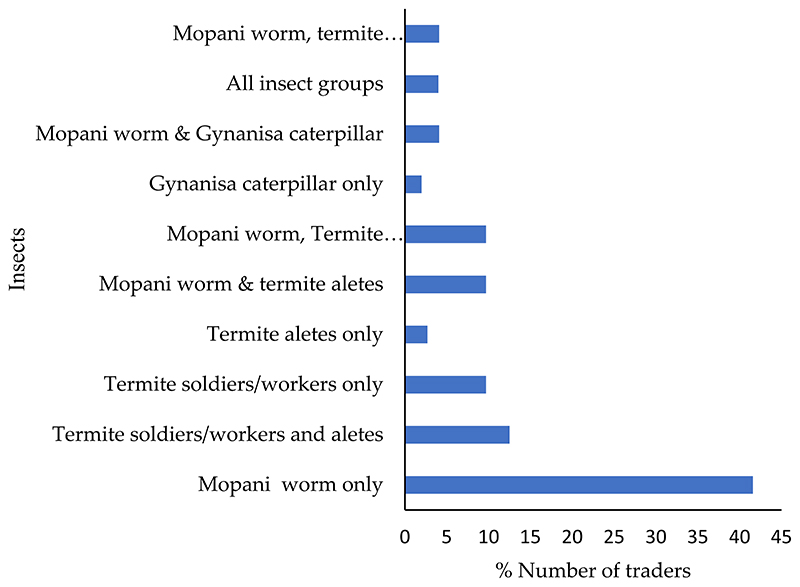
Edible insects sold in various towns of the Vhembe district, Limpopo province, South Africa (n = 72). All species refers to mopani worms, termite soldiers or workers and alates, Gynanisa caterpillars, grasshoppers, and stinkbugs.

Of the traders, 42% only sold mopane worms, followed by traders that sold a combination of termites (13%), and traders that sold a combination of mopani worm and termites (10%). In contrast, fewer traders (2%) only sold Gynanisa caterpillars (Figure 1).

There were significant differences in the number of traders who sold mopane worms (X^2^_7_=23.996, *p* < 0.05), termite soldiers or workers (X^2^_7_ = 60.484, *p* < 0.05), termite alates (X^2^_7_ = 41.203, *p* < 0.05), and Gynanisa caterpillars (X^2^_7_ = 15.707, *p* < 0.05) across towns, but there were no significant differences in the number of traders who sold stinkbugs (X^2^_7_ = 22.242, *p* > 0.05) or grasshoppers (X^2^_7_ = 6.105, *p* > 0.05).

Stinkbugs and grasshoppers were out of season and not seen in the markets during the survey. However, our analysis was based on the responses of traders who sell stinkbugs and grasshopper when they are in season. Of the traders, 94% bought insects from middlemen, with only 6% involved in insect harvesting from the wild.

**Figure 2:**
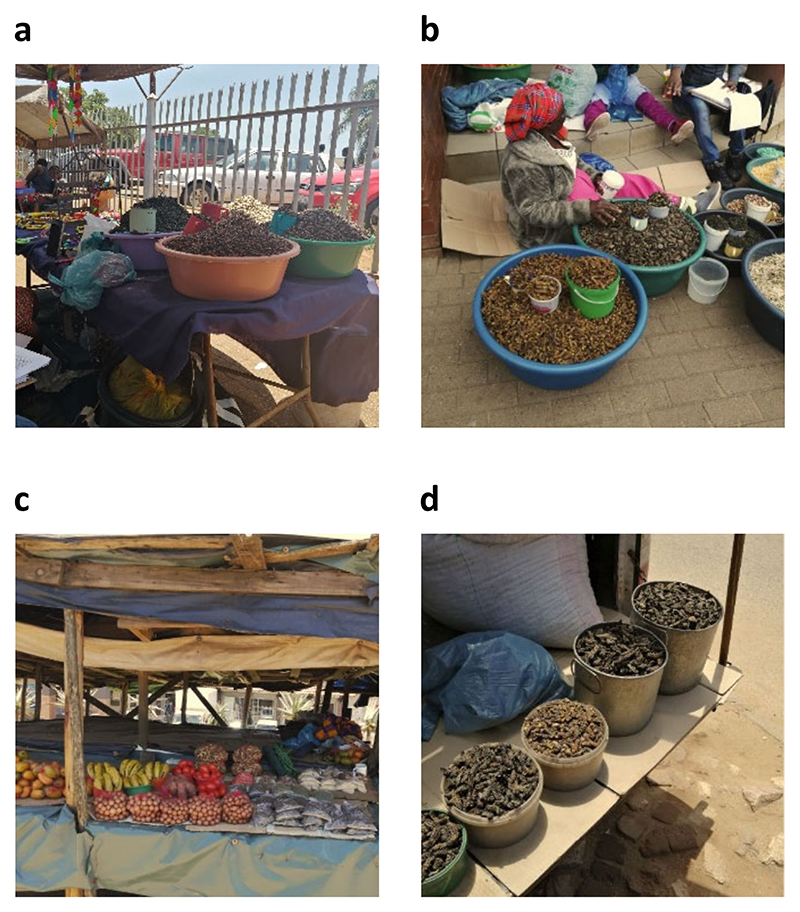
Conditions of informal markets where edible insects are sold in the towns of Vhembe district, South Africa: (**a**) Thohoyandou, (**b**) Makhado, (**c**) Elim, and (**d**) Sibasa.

Insect trading takes place in informal markets along the street, pavements, and on table stalls composed of cardboard and wood. The majority of trading stalls had no protection from the sun (Figure 2). Some traders were sitting down on pavements along roadsides. Safety and hygiene were the major issues of concern stated by the respondents in the Vhembe district. Informal markets were untidy with few garbage disposal units. There were no dedicated sanitation services for traders (including running water and toilets). The majority of the traders had no secure storage units in which to put their stock; as a result, several traders were forced to travel with their stock every day because of the high theft rate.

**Figure 3:**
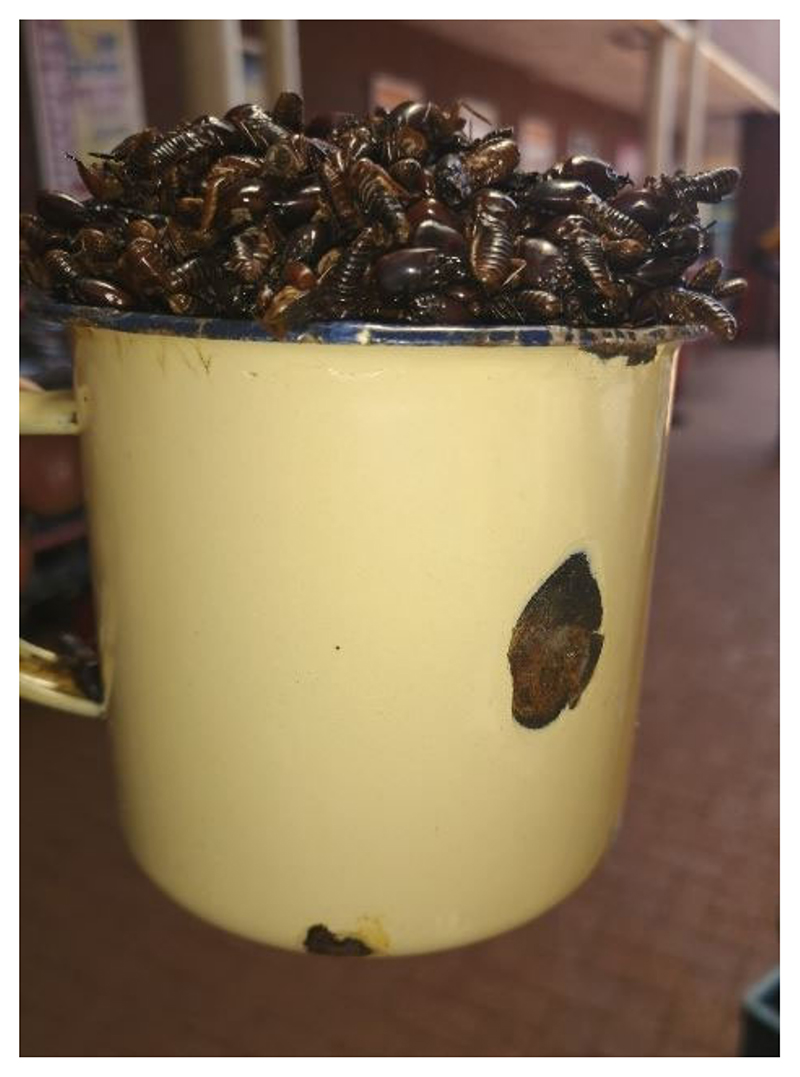
Steel cup (200 g) used to measure single serving of insects, in this case, termites.

The retail price of edible insects per steel cup (200 g) varied (caterpillars: ZAR 30 (USD 1.85) per cup; termites: ZAR 20 (USD 1.23) per cup (see Figure 3)). Traders in the area usually buy a 5 kg bucket of the caterpillars costing ZAR 300 (USD 18.5) from the middle-man, which generates ZAR 2000–3000 (USD 123–185) for the trader. In addition, a 50 kg sack of caterpillars costs ZAR 3000 (USD 185) and generates a total of ZAR 15,000 (USD 923). On the other hand, a 5 L bucket of termites costs ZAR 200 (USD 12.3) from the middleman and generates an income of ZAR 1500 (USD 92.3).

**Figure 4:**
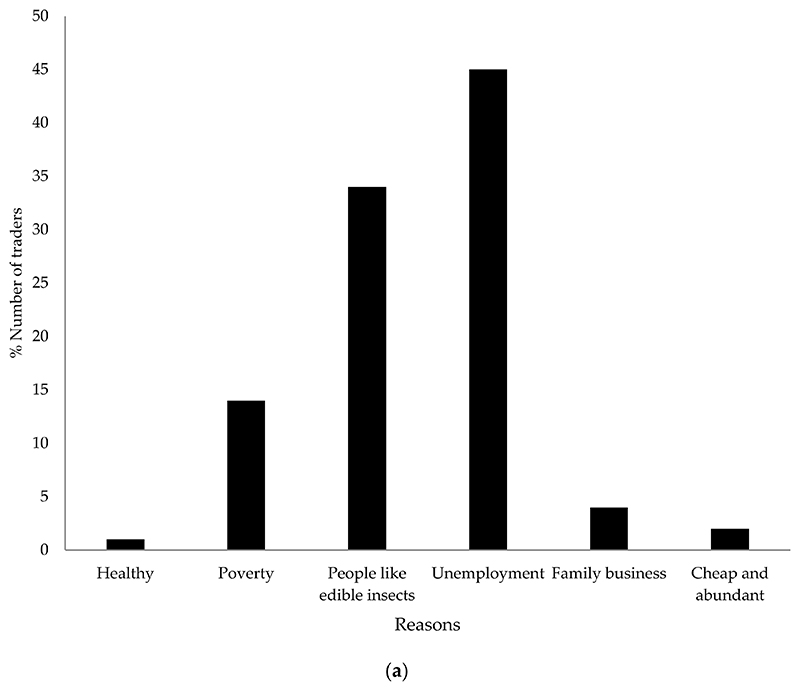

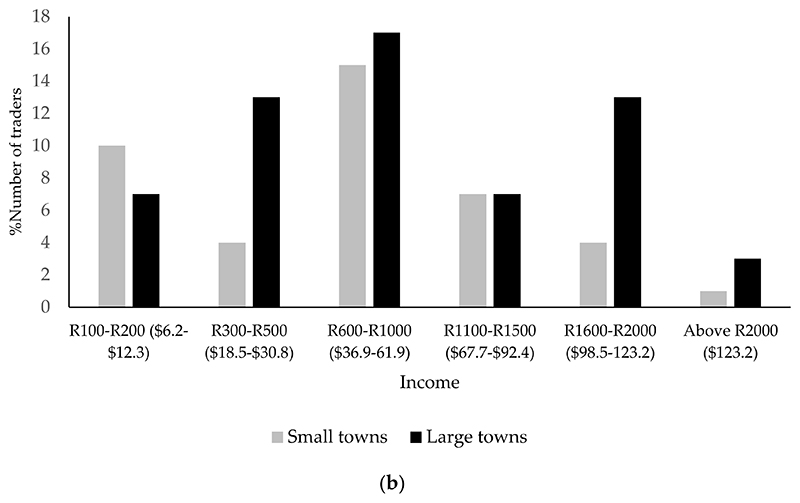
(**a**) Reason for trading insects and (**b**) weekly profit generated by traders from selling edible insects across small towns (i.e., Elim, Sibasa, Tshakuma, Biaba and Halambani) and large towns (i.e., Thohoyandou, Makhado and Musina) in Vhembe district (*n* = 72 traders).

Of the traders, 45% indicated that unemployment was the primary reason for selling edible insects. The second (34%) primary reason for trading was the increased demand for edible insects by consumers, while 14% cited poverty. Few (1%) traders indicated that they sold insects because of their health benefits (Figure 4a).

Most traders (60%) cited that trading insects provided financial support, while 35% cited that they use money generated from trading insects to pay school fees and electricity, and buy household items. However, 5% mentioned that they do not benefit much from selling insects. Of the traders, 3% earned over ZAR 2000 (USD 123) per week in large towns, while only 1% of the traders earned over ZAR 2000 (USD 123) per week in small towns (Figure 4b).

**Figure 5:**
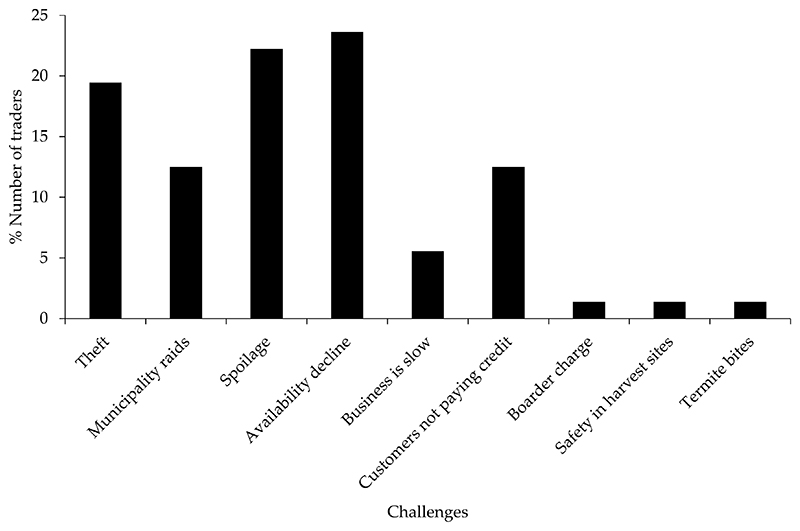
Challenges associated with trading in insects in Vhembe district, Limpopo.

Most traders (24%) cited that the decline in the availability of edible insects from the wild and insect spoilage (22%) were the major challenges associated with trading insects. Fewer traders (1%) cited safety and termite bites as challenges associated with trading insects (Figure 5).

A total of 36 policy documents from various governmental organizations in Limpopo were reviewed, and only 4 included edible insects in economic development strategies. Only the mopane worm was included in economic development strategies (Table 1). Interventions were aimed at providing infrastructure for processing, packaging, storage, etc.; however, only one plan provided a budget for this (Table 1).

## 4 Discussion

Edible-insect trading is an everyday activity that provides seasonal employment for unemployed communities in Vhembe district, particularly for females [12]. This study found that almost all the traders were women, and when men did trade, they were over 35 years of age. Insect trading is primarily female-driven in most countries in Africa [12,25–27]. These results indicate that women play an essential role in rural livelihoods, and they are the back-bone of subsistence household food security. Women depend on edible insects to generate income, which reduces poverty and improves household food security [28–30]. Findings from the current study revealed that trading in insects provides earning opportunities for women in rural areas of Vhembe district in Limpopo province, South Africa. Similar findings were reported by Netshifhefhe et al. [12], who stated that trading in termites in Vhembe district, Limpopo Province created employment opportunities for women in this district. Similarly, Makhado et al. [7] reported that trading in insects provided earning opportunities in the Greater Giyani Municipality in Limpopo. Edible-insect trading creates opportunities for women to participate in economic development in rural areas, thus empowering them with income opportunities and improving their livelihoods [27,28,31]. This directly contributes to SDG 1, 2, and 5 of no poverty, zero hunger, and achieving gender equality and empowerment for all women and girls, respectively [27]. The National Development Plan of South Africa’s goal is to eradicate poverty and to reduce inequality by 2030. Therefore, expanding edible-insect markets can contribute to realizing this goal. Edible insects should be adopted as an economic development strategy to end poverty in developing countries and empower women by providing earning opportunities.

Edible insects are an important natural resource used as food and cash income in southern Africa [7,21,32]. Mopane worms were the most sold insects across towns in the Vhembe district. Mopane worms are a popular food source and a delicacy that is widely consumed in southern Africa [21,24]. The sale of mopane worms results in an income of approximately USD 39 million to 59 million per annum across South Africa, Zimbabwe, and Botswana [20,24]. Edible insects provide earning opportunities that play a crucial role in improving livelihoods and reducing poverty in rural areas [12,24,30]. The consumption and trading of edible insects are essential coping strategies contributing to household food security and nutrition [24].

Edible insects play an important role in sustaining rural livelihoods [16,32]. Harvesting and selling insects improve rural livelihoods by providing an income that is used for basic needs such as paying for school fees, food, and electricity bills [33,34]. Trading edible insects provides seasonal employment and creates earning opportunities in rural communities in Southern Africa [7,35]. Generated income from trading insects in Vhembe district, Limpopo is in the range of ZAR 100–200 per week to over ZAR 2000 per week. These findings are in line with those of Netshifhefhe et al. [12], who reported that the income from selling termites in Limpopo is, on average, ZAR 292 (USD 18) a day, ZAR 2395 (USD 147) a month, and ZAR 20400 (USD 1256) a year. On the other hand, in Namibia and Cameroon, income from selling insects was estimated to be USD 71–4800 per season [26,36]. Agea et al. [33] reported that traders said the income from selling insects had improved their standard of living [33].

South Africa remains one of the countries with the highest unemployment rate globally [37–39], and unemployment is a significant socioeconomic issue facing this country. According to Statistics South Africa [40], the unemployment rate in South Africa was estimated to be 30.1% in the first quarter of 2020, and approximately 7.1 million South African were unemployed in that quarter. Most of the unemployed population in South Africa is in rural areas [38]. The majority of the population in Limpopo live in rural areas, and this province is the third poorest province in South Africa, with 21.8% of the population living under poverty [40,41]. Unemployment and poverty are the primary reasons for trading insects in the Vhembe district. According to Makhado et al. [7], 82% of the respondents in 6 villages of the Greater Giyani Municipality, Limpopo are unemployed and dependent on woodlands natural resources such as edible insects, wild fruits and herbs, medicinal plants, and fuelwood for household nutrition and income generation. The unemployment rate in Limpopo was 49.9% in 2020; therefore, expanding and promoting the edible-insect business in this province would create job opportunities and reduce poverty [40].

Edible insects are a traditional food source that has been consumed in South Africa for centuries [42,43]. However, edible insects are still not included in food policies and legislation that govern the use of insects as food [44]. Edible insects are not regulated in terms of food safety and quality, even though they are widely consumed in Limpopo and in some parts of Mpumalanga, Gauteng, North West, and KwaZulu-Natal provinces [11,45,46]. Both the government and nongovernmental organizations are not doing enough to protect, promote, and encourage the culture of consuming insects in South Africa. Edible insects are seen as food for poor people, dirty, and undignified [47]. For example, edible insects are mostly (rarely anywhere else) sold in informal markets where selling conditions do not meet the food standards requirements approved by the Departments of Health; of Agriculture, Forestry, and Fisheries; and Trade and Industry [44]. The informal markets where insects are sold have no proper sanitation (running water and toilets) and storage facilities and are fundamentally unhygienic and unsafe.

Little is done to improve the conditions where edible insects are sold, as indicated by our assessment of municipal development plans. Trading conditions are poor and unhygienic with no proper sanitation. This shows a lack of respect and human dignity, which is unconstitutional given Section 10 of the South African constitution: “Everyone has inherent dignity and the right to have their dignity respected and protected” (Constitution of the Republic of South Africa, 1996). Important cultural practices should be celebrated and embraced by the government (in terms of Sections 30 and 31 of the Constitution), by offering support such as infrastructure, proper storage facilities, and funding to improve the trading conditions of edible insects. This would also ensure that food safety and hygienic practices are met. The personal circumstances and needs of the traders and their clients should be accorded the necessary dignity and respect.

Edible insects are rarely included in rural development strategies. Few governmental organisations included edible insects in rural development strategies in Limpopo. Therefore, resources and budget to develop and expand the edible insects sector are limited [48]. The lack of clear legislation and regulatory guidelines governing trading and insect use as food in South Africa might be the primary barrier that prevents local authorities from including edible insects in rural economic development plans [44]. In addition, the lack of legislation and regulatory guidelines that recognise and govern the edible insect sector as part of agricultural activity is concerning [49]. There are no clear guidelines on the use of insects as food in South Africa. There are no standards on the use of insects for human consumption [44]. Food laws, such as the Meat Safety Act (2000), and policies in South Africa do not include insects, and insects have not been tested and approved for human consumption [44]. This may be the reason for not including edible insects in economic development strategies. The National Biodiversity Economic Strategy [50] does not include developing insects in rural areas. In addition, this might be the major barrier preventing the expansion of the edible-insect food sector in South Africa and other parts of the world [51].

Governmental and local authorities are aware of the potential of edible insects in addressing food insecurity and poverty in South Africa. For example, the Integrated Development Plan 2020/2021 of the Greater Giyani local municipality recognised mopani worm trading as an important local economic development activity that contributes to livelihoods in this municipality. Furthermore, the Greater Giyani Municipality Local Economic Development Strategy of 2014–2016 had plans to establish a mopane worm processing centre to be used by traders to hygienically sell and store large quantities of mopani worms [52]. This municipality also wanted to expand the mopani worm value chain to neighbouring SADC countries. This initiative provides job opportunities to local people in Giyani, South Africa [52]. The local Musina municipality also wanted to establish a mopani worm production facility used for storing and processing mopani worms by local traders and harvesters [53]. The Greater Giyani and Musina local municipalities are moving in the right direction by including mopani worms in their local economic development strategies, but these strategies need to translate into implementation on the ground, which requires the allocation of a necessary budget. The focus should not be on mopani worms; other edible insects (for example, termites, stink bugs and grasshoppers) that are consumed in these municipalities should be prioritised. Other municipalities should also include edible insects in their local economic development strategies because they can create earning opportunities for rural communities.

The decline in availability of edible insects is the main challenge faced by traders in Vhembe district. Edible insects are collected in the wild, and their availability is affected by climate change, uncontrolled harvesting, and land use changes such as clearing of land and agriculture [44,46]. The decline in the availability of insects affects rural communities who depend on edible-insect trading for a living. As a result, edible insects could be farmed to ensure long-term availability and accessibility when the natural supply is limited [54,55]. Edible insects are successfully farmed in Thailand, Kenya, Uganda, Ghana, and Zimbabwe [51,56]. The Flying Food project in Kenya and Uganda are small-scale insect-farming projects that successful farmed edible crickets [57], while the Aspire Food Group project specializes in farming palm weevils in Ghana [58]. Mopane Worm Enterprises from Zimbabwe specializes in farming mopane worms [59]. These small-scale insect farms are good examples of successful insect farming that can be adopted in South Africa, thereby creating localised value chains. Farming edible insects reduces overharvesting pressure and ensures that edible insects contribute to rural development, job creation, and social wellbeing without putting pressure on the species in the wild. However, there must be clear legislation and regulatory frameworks governing the farming of edible insects to ensure that proper standards and guidelines are followed [49,51]. Insect farming in South Africa can provide training opportunities and skills that would empower rural communities.

## 5 Conclusions

Edible insects are a traditional food source that plays a vital role in rural food security. Traditional food primarily contributes to rural livelihood improvement as a source of income and nutrition. This study determined the socioeconomic benefits of trading insects in the Vhembe district, South Africa. Trading in edible insects is an economic activity that creates earning opportunities for unemployed people in rural areas, especially women. As a result, edible-insect trading plays an essential role in alleviating poverty for unemployed rural people in Vhembe district. Mopane worms were the most sold insect in various towns across the district. One respondent said that mopani worms are desired by the consumers and often attract customers to buy, which improves business for insect traders. Edible insects could generate income for many more people in rural areas. For example, in the Asia-Pacific region (Thailand, Cambodia, and China), edible-insect farming is well-established and plays an essential role in alleviating poverty in rural areas [60]. The market of edible insects in this region is expected to exceed USD 270 million by 2024 [60,61]. In addition, the global edible-insect market is projected to reach USD 4.63 billion by 2027 [62]. Therefore, edible insects should be included in food policies and prioritised as a nutritious food source that can improve food and nutrition security in South Africa. The associated education and awareness interventions are necessary to promote their use. Edible-insect trading can be adopted as a rural development and poverty eradication strategy to improve rural livelihoods and create job opportunities. However, traders should be provided with infrastructure and funding for insect farming as part of the rural development program to create job opportunities while empowering rural communities, thus contributing to food security. Edible-insect farming ensures the accessibility and growth of edible-insect markets in rural areas. In addition, this could provide stable earning opportunities for insect traders and farmers. Edible insects can generate revenue that can contribute to the rural economy, thereby reducing poverty in rural areas. Government officials should invest in expanding the sale of edible insects in the Vhembe district, because they play an important role in generating income for traders in this district.

## Supplementary Material

Supplementary Material

## Figures and Tables

**Table 1:** Policy documents that included edible insects in economic development strategies in various governmental organisations in Limpopo province.

Municipality	Policy Document	Year	Edible Insects Included	Action/Explanation	Budget Allocation
Limpopo (Economic Development Agency)	Strategic plan	2020–2025	None	No action	
Limpopo (Provincial treasury)	Strategic plan	2020–2025	None	No action	
Vhembe district Municipality	IDP	2020–2021	None	No action	
Musina local municipality	IDP	2020–2021	None	No action	
Musina local Municipality	IDP	2015–2016	Mopani worm	No action	
Musina local Municipality	IDP	2013–2014	Mopani worm	No Action	
Musina local Municipality	LED	2018	Mopani worm	Planned to establish mopani worm production facility for processing and packaging of mopani worm	
Collins Chabane local Municipality	IDP	2017–2018	None	No action	
Collins Chabane local Municipality	IDP	2018–2019	None	No action	
Collins Chabane local Municipality	IDP	2019–2020	None	No action	
Collins Chabane local Municipality	IDP	2020–2021	None	No action	
Thulamela local Municipality	IDP	2020–2021	None	No action	
Makhado local Municipality	LED	2013	None	No action	
Mopani District Municipality	IDP	2016–2021	None	Recognises mopani worm as an important economic activity in the district	
Mopani District Municipality	IDP	2006–2012	None	No action	
Greater Giyani local Municipality	IDP	2020	Mopani worm	The municipality recognizes mopani worms trading as an important economic activity. Dzumeri Processing centre used by insect traders and harvesters to process, store and trade mopani worm	
Greater Giyani local Municipality	LED	2014–2016	Mopani worm	Greater Giyani Natural Resource program aimed at expanding mopani worm business. The municipality proposed building a processing center used by local traders to store large quantities of mopani worms and expand mopani worm value chains to the neighboring SADC countries.	R11.6 million ($801104.97)
Greater Tzaneen Municipality	LED	2018	None	No action	
Greater Tzaneen Municipality	IDP	2015–2016	None	No action	
Greater Tzaneen Municipality	IDP	2019–2020	None	No action	
Capricon District Municipality	IDP	2016–2021	None	No action	
Blouberg local Municipality	IDP	2015–2016	None	No action	
Blouberg local Municipality	IDP	2019–2020	None	No action	
Blouberg local Municipality	IDP	2018–2019	None	No action	
Blouberg local Municipality	IDP	2020–2021	None	No action	
Molemole local Municipality	LED	2012	None	No action	
Molemole local Municipality	LED	2019–2024	None	No action	
Lepelle-Nkumpi local Municipality	IDP	2015–2016	None	No action	
Lepelle-Nkumpi local Municipality	IDP	2016–2021	None	No action	
Lepelle-Nkumpi local Municipality	IDP	2017–2018	None	No action	
Lepelle-Nkumpi local Municipality	IDP	2015–2016	None	No action	
Lepelle-Nkumpi local Municipality	IDP	2020–2021	None	No action	
Lepelle-Nkumpi local Municipality	LED	2019	None	No action	
Aganang local Municipality	LED	2013	None	No action	
Aganang local Municipality	IDP	2009–2010	None	No action	
Polokwane local Municipality	IDP	2019–2020	None	No action	
Polokwane local Municipality	IDP	2021–2021	None	No action	

IDP, Integrated Development Plan; LED, Local Economic Development strategy. Makhado and Musina local municipalities fall under Vhembe District Municipality, and Blouberg under Capricorn District Municipality, and Greater Giyani and Greater Tzaneen under Mopani District Municipality.

## Data Availability

Data presented in this study are available in the tables, figures, and appendices of the current manuscript.
