# Supplementary material for: The Role of Edible Insects in Rural Livelihoods, and Identified Challenges in Vhembe District, Limpopo, South Africa
Source: Resources (Basel). Author manuscript; Available in PMC 2023 Sep 15. (PMC7615094; doi:10.3390/resources10120123)
Supplement: Supplementary Material [file EMS186859-supplement-Supplementary_Material.pdf]

Supplementary Materials: The following are available online at https://www.mdpi.com/article/10.3390/resources10120123/s1, File S1. Questionnaire to Source Information from Traders in Vhembe District, Limpopo.

Appendix A

Table A1. Demographic information of traders across towns in Vhembe district (*n* = 72).

Demographics	Elim	Louis Trichardt	Musina	Sibasa	Thohoyandou	Tshakhuma
Age category (years)						
Under 18	0	0	0	0	0	0
18–24 years	4	0	0	0	2	0
25–34	0	0	0	1	3	3
35–44	2	1	4	2	7	6
45–54	0	4	2	1	5	4
55–64	1	1	1	5	6	1
65–74	3	0	1	0	4	0
75 years and above						
Sex						
Male	1	0	1	1	1	0
Female	7	6	7	8	26	14
Level of education						
No formal education	1	0	0	0	0	0
Primary education	1	3	2	1	9	0
Secondary education	4	3	6	6	17	14
Tertiary education	0	0	0	2	0	0
Occupation						
Unemployed	0	0	0	0	1	0
Self employed	5	6	8	8	25	14
Pensioner	3	0	0	0	1	0
Employed	0	0	0	0	0	0
Student	0	0	0	0	0	0
